# Purification and Characterization of a New CRISP-Related Protein from *Scapharca broughtonii* and Its Immunomodulatory Activity

**DOI:** 10.3390/md18060299

**Published:** 2020-06-04

**Authors:** Wanying Liu, Sixue Bi, Chunlei Li, Hang Zheng, Zhongyi Guo, Yuanyuan Luo, Xiaozheng Ou, Liyan Song, Jianhua Zhu, Rongmin Yu

**Affiliations:** 1 Biotechnological Institute of Chinese Materia Medica, Jinan University, Guangzhou 510632, China; lwy5464@stu2017.jnu.edu.cn (W.L.); zh923205294@163.com (H.Z.); 2 Department of Pharmacology, College of Pharmacy, Jinan University, Guangzhou 510632, China; shuimuliunian@163.com (S.B.); joey444@163.com (Z.G.); geminijane@126.com (X.O.); tsly@jnu.edu.cn (L.S.); 3 Department of Natural Product Chemistry, College of Pharmacy, Jinan University, Guangzhou 510632, China; lcl992@126.com (C.L.); yyluooo@163.com (Y.L.)

**Keywords:** *Scapharca broughtonii* protein, purification, structural characterization, CRISP-related protein, immunomodulatory activity

## Abstract

More and more attention has been paid to bioactive compounds isolated from marine organisms or microorganisms in recent years. At the present study, a new protein coded as HPCG2, was purified from *Scapharca broughtonii* by stepwise chromatography methods. The molecular weight of HPCG2 was determined to be 30.71 kDa by MALDI-TOF-MS. The complete amino acid sequence of HPCG2 was obtained by tandem mass spectrometry combined with transcriptome database analysis, and its secondary structure was analyzed using circular dichroism. HPCG2 comprised 251 amino acids and contained 28.4% α-helix, 26% β-sheet, 18.6% β-turn, and 29.9% random coil. HPCG2 was predicted to be a cysteine-rich secretory protein-related (CRISP-related) protein by domain prediction. Moreover, HPCG2 was proved to possess the immunomodulatory effect on the murine immune cells. MTT assay showed that HPCG2 promoted the proliferation of splenic lymphocytes and the cytotoxicity of NK cells against YAC-1 cells. Flow cytometry test revealed that HPCG2 enhanced the phagocytic function of macrophages and polarized them into M1 type in RAW264.7 cells. In particular, Western blot analysis indicated that the immunomodulatory mechanism of HPCG2 was associated with the regulation on TLR4/JNK/ERK and STAT3 signaling pathways in RAW 264.7 cells. These results suggested that HPCG2 might be developed as a potential immunomodulatory agent or new functional product from marine organisms.

## 1. Introduction

The immune system is an important system for the body to perform immune responses and functions. The occurrence of many diseases is closely related to immune disorders or immune deficiency. The immune organs are very important for host immune responses [1]. For example, the spleen is the most important organ for antibacterial and antifungal immune activities [2]. T and B lymphocytes and natural killer (NK) cells are the largest cell types in the spleen. NK cells are effector lymphocytes that control several types of tumor and microbial infection by limiting their spread and subsequent tissue damage [3]. Macrophages are vital members of the immune system which are derived from monocytes. In the immune system, they are used to defend against the invasion of foreign substances or to clear damaged cells. At the same time, macrophages are also involved in some important biological reactions, such as tumor resistance, embryonic development, and lung function maintenance [4]. Depending on the different growth environments, macrophages may polarize into the M1 or M2 type. M1 macrophages are classically activated by Th1 cytokines (interferon [IFN]-γ). The expression of surface molecule CD86 and major histocompatibility complex II (MHC II), as well as the secretion of cytokine tumor necrosis factor-α (TNF-α) and toxic molecules like nitric oxide are elevated by M1 macrophages, which are typically described as tumor-killing macrophages. M2 macrophages, alternatively induced by Th2 cytokines (interleukin [IL]-4 and IL-13), act as a powerhouse for tumor angiogenesis and metastasis. Most of the macrophages in the tumor microenvironment manifested as an M2-like phenotype, which facilitates the immunological tolerance and tumor progression microenvironment [5]. Based on this situation, immune modulators are often used clinically to regulate the immune function of the body. 

In recent years, marine natural products have been proved to be effective biological regulators which possess antitumor, antibacterial, antioxidant, and immunomodulatory activities, etc. [6]. More and more attention has been paid to bioactive compounds isolated from marine organisms or microorganisms. Many of the marine-derived proteins, peptides, and protein hydrolysates can significantly affect the immune system function on multiple levels, such as directly or indirectly inducing chemotaxis of immune cells, regulating cell differentiation, and inhibiting excessive inflammation. In addition, compared with synthetic immune modulators, natural immune modulators are considered to have a mild effect and lower side effects [7,8].

*Scapharca broughtonii* (*Arca inflata* Reeve), mainly distributed in the western Pacific and Indian Ocean coast, is an economical shellfish that has been industrially farmed [9]. Being rich in proteins, *S. broughtonii* has been used as a nutritious food all over the world and a kind of medicinal material in East and South Asia. To date, there have been several studies performed on the active protein components in *S. broughtonii*. Sb-BDef1, a big defensin characterized from *S. broughtonii*, was proved to be a consecutive expressing protein and could be induced by *Vibrio anguillarum* infection [10]. The cDNA of a novel Sb-BPI/LBP1 was identified and its expression level was significantly upregulated by lipopolysaccharide (LPS) stimulation [11]. SbMnSOD, an antioxidant and antibacterial defense enzyme, was also observed in *S. broughtonii* and expressed in fusion form [12]. 

In our previous research, several bioactive proteins possessing antioxidant, antitumor, and antimicrobial activities were isolated and characterized from *S. broughtonii* [13,14,15]. However, the proteins with immunoregulatory activity have not been fully studied. Unlike vertebrates, the innate immune response of invertebrates such as *S. broughtonii* occurred in the hemolymph [16]. Therefore, it is likely to discover proteins with immunomodulatory activity in the hemolymph of *S. broughtonii*.

As a part of our serial research in *Scapharca* species, a new protein named HPCG2 from the hemolymph of *S. broughtonii* was isolated and purified in the present study. The in vitro immunomodulatory activity of HPCG2 was evaluated as well. This work will be helpful to understand the role of the hemolymph in the immune defense of *S. broughtonii* and demonstrate the possibility of developing new immune-enhancing agents or functional products from marine organisms.

## 2. Results

### 2.1. Purification of HPCG2

**Figure 1:**
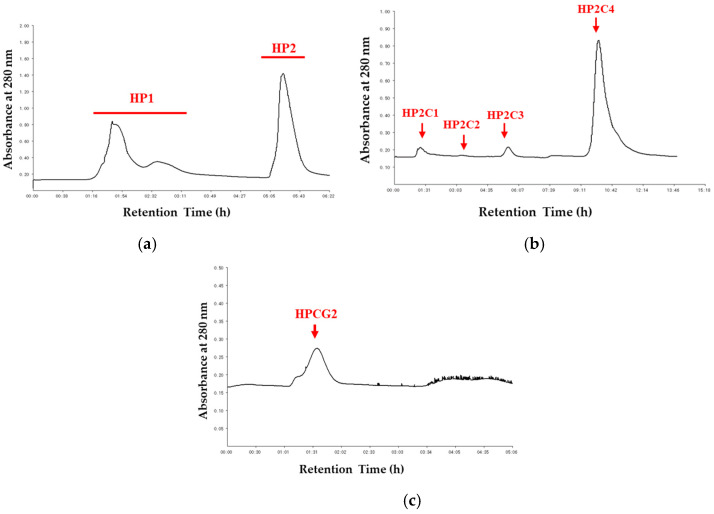
Stepwise chromatography purification of HPCG2. (**a**) elution profile of HP by DEAE Sepharose fast flow column; (**b**) elution profile of HP2 by Phenyl Sepharose CL-4B column; (**c**) elution profile of HP2C3 by Sephadex G-75 column.

**Figure 2:**
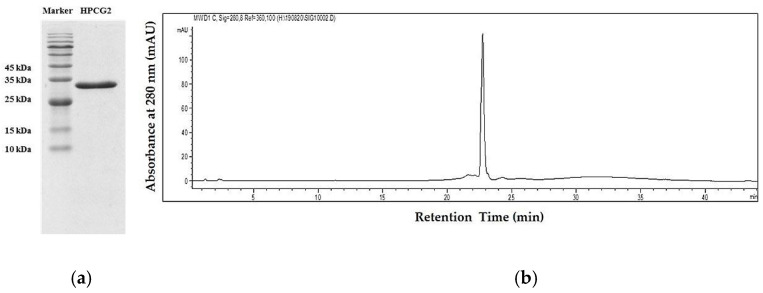
Purity verification of HPCG2. (**a**) SDS-PAGE of HPCG2; (**b**) RP-HPLC of HPCG2.

The salting-out method was selected to extract the crude protein from *S. broughtonii* materials with minor modifications. As displayed in Figure 1 and Figure S1, HPCG2 was obtained through column chromatographic procedure combined with SDS PAGE analysis. The protein concentration of HPCG2 was finally determined as 97.14 ± 0.56%, and no carbohydrate was detected. In addition, HPCG2 showed a single band around 30 kDa in sodium dodecyl sulfate polyacrylamide gel electrophoresis (SDS-PAGE) analysis and a symmetrical peak with the purity over 95% in RP-HPLC analysis (Figure 2). These results revealed HPCG2 was a monomeric, homogeneous protein without carbohydrates.

### 2.2. Structural Characterization of HPCG2

**Figure 3:**
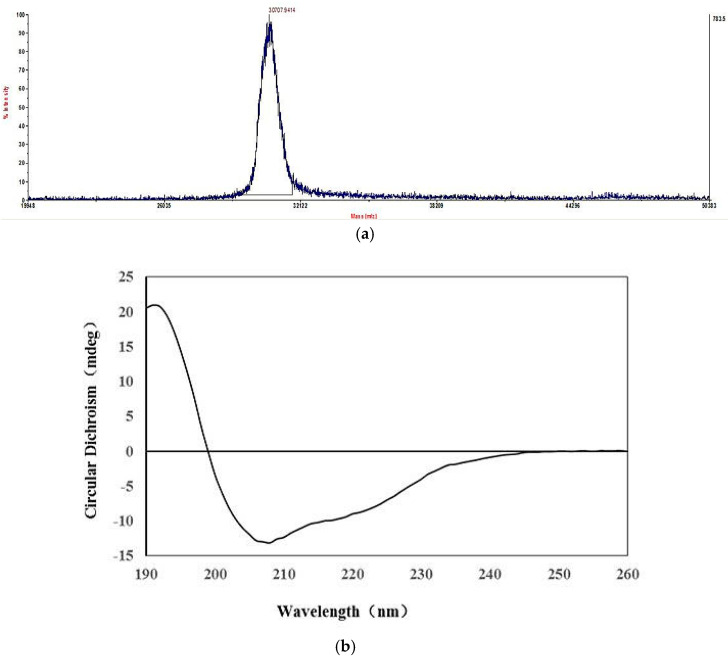
Characterization of HPCG2. (**a**) Molecular weight of HPCG2 measured by MALDI-TOF-MS; (**b**) CD spectrum of HPCG2.

Mass spectrometry is a powerful tool in the field of protein research for protein identification, quantification, and analysis [17]. The accurate molecular weight was measured by matrix-assisted laser desorption/ionization time-of-flight mass spectrometry (MALDI-TOF-MS) as 30.71 kDa (Figure 3a), which was consistent with the result of SDS-PAGE.

Circular dichroism (CD) is a fast and accurate method for the study of protein conformation in a dilute solution. It was reported that the α-helical structure displayed a positive band near 192 nm and two negative characteristic shoulder bands at 222 and 208 nm. The spectrum of β-sheet had a negative band at 216 nm, and a positive band between 185 and 200 nm. There was a positive CD band around 206 nm in the spectrum of β-turn structure [18,19]. As shown in Figure 3b, the CD spectrum of HPCG2 exhibited a strong positive band around 195 nm and two negative shoulder bands near 208 and 220 nm, which indicated the existence of α-helical and β-sheet. CD analysis using a Neural Networks (CDNN) program was used to calculate the content of each secondary structure in HPCG2, and the results were shown in Table 1. The total content of regular secondary structure types was approximately 70%, which indicated that HPCG2 had an ordered conformation [20].

**Figure 4:**
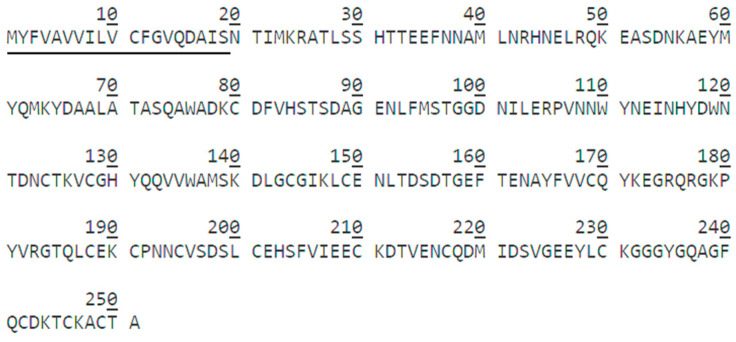
Amino acid sequence of HPCG2. The signal peptide predicted by SignalP 4.1 server was underlined.

**Figure 5:**
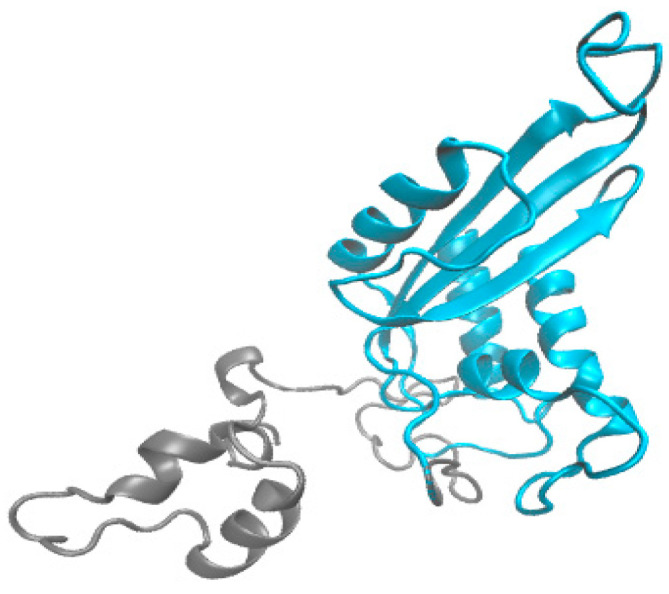
Inferred tertiary structure of HPCG2. A snake venom CRISP from *N. atra* (PDB number: 1XTA) was used as a template and the CAP domain was displayed in blue.

In this study, tandem mass spectrometry was used to identify the complete amino acid sequence of HPCG2. HPCG2 was digested by trypsin and productive peptides, detected and aligned with the protein sequence database from the transcriptome of *S. broughtonii* (Table S1). As illustrated in Figure 4, HPCG2 comprised 251 amino acids and had a theoretical isoelectric point of 4.81. The tertiary structure was inferred by I-TASSER server based on amino acid sequence of HPCG2 (Figure 5). The model was built using the protein named Natrin, a snake venom CRISP from *Naja atra* (PDB number: 1XTA). The C-score and TM-score of the model were −0.84 and 0.61 ± 0.14, which indicated the high quality of the predicted model [21].

### 2.3. Sequence Analysis of HPCG2

Protein signature prediction of HPCG2 illustrated a widespread domain named CAP (the cysteine-rich secretory proteins, antigen 5, and pathogenesis-related 1 proteins), and HPCG2 was classified as a cysteine-rich secretory protein-related (CRISP-related) family within the CAP superfamily (Figure S2). 

**Figure 6:**
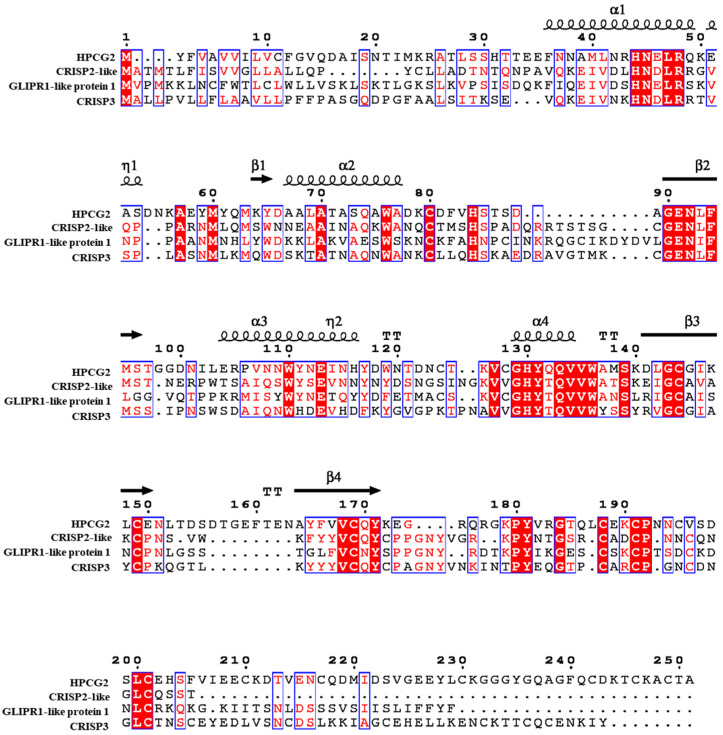
Sequence alignment between HPCG2 and other proteins of CRISP-related family. Identical amino acid residues were in the red background. Amino acid residues with 75% identity were marked by boxes. (Accession numbers and sources of proteins: XP_010885778.1 [*Esox lucius*], XP_028728901.1 [*Peromyscus leucopus*], O19010.1 [*Equus caballus*]).

The homology analysis in the NCBI database was performed using the PSI-BLAST program based on the amino acid sequence. As shown in Figure 6, HPCG2 had certain homology with proteins from CRISP-related family, such as CRISP2-like (XP_010885778.1, 37.02%), GLIPR1-like protein 1 (XP_028728901.1, 36.18%), and CRISP3 (O19010.1, 35.21%). They all have the signal peptide at the N-terminal, which was consistent with the characteristics of most members of CRISP-related family [22]. The result also indicated the consistency of these proteins in the distribution of secondary structure. Based on the above analysis, HPCG2 could be identified as a CRISP-related protein.

### 2.4. Effects of HPCG2 on Splenic Lymphocyte Proliferation and NK Cell Cytotoxicity

**Figure 7:**
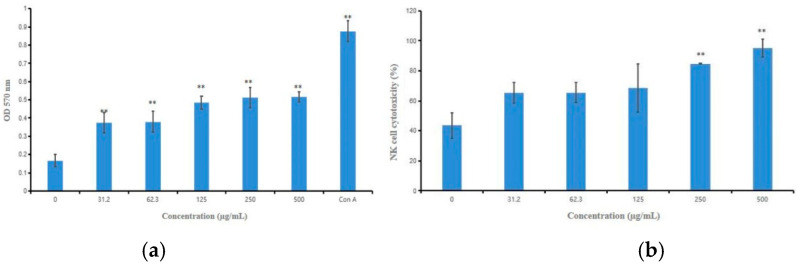
Effects of HPCG2 on splenic lymphocyte proliferation and NK cell cytotoxicity. (**a**) effect of HPCG2 on splenic lymphocyte proliferation; (**b**) effect of HPCG2 on NK cell cytotoxicity to YAC-1 cells. Values are expressed as mean ± SD obtained from triplicate experiments (*n* = 3). Statistical analysis was performed by an independent *t*-test using the statistical analysis software SPSS 11.5. ** *p* < 0.01 versus the negative control.

The spleen is the most important organ for antibacterial and antifungal immune reactivity, and it mediates the innate and adaptive immune reaction [1]. Immunomodulatory activities of HPCG2 were appraised by determining the splenic lymphocyte proliferation. Figure 7a showed that HPCG2 significantly promoted the proliferation of splenic lymphocytes with increasing HPCG2 concentrations. 

Meanwhile, we detected the cytotoxic activity of NK cells treated by HPCG2 against YAC-1 cells. As shown in Figure 7b, HPCG2 strengthened the killing activity of NK cells to YAC-1 cells in vitro (*p* < 0.05, *p* < 0.01) at concentrations of 250 and 500 µg/mL.

### 2.5. Effect of HPCG2 on the Viability of RAW264.7 Macrophages

**Figure 8:**
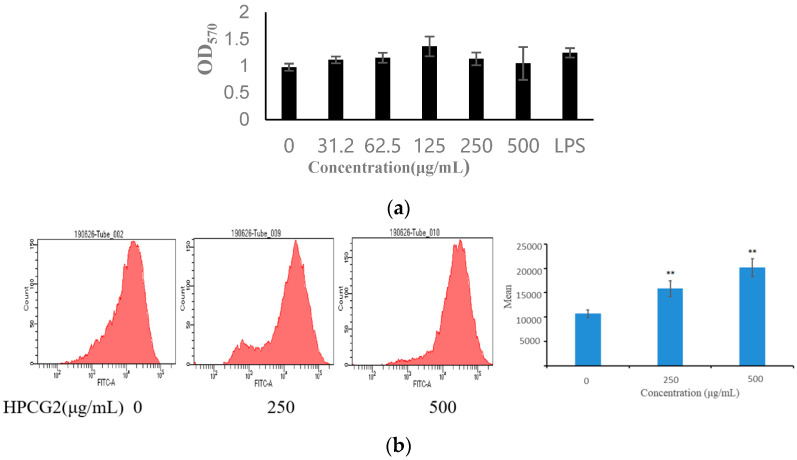
Effects of HPCG2 on the viability and phagocytosis function of RAW264.7 cells. (**a**) effect of HPCG2 on the vitality of RAW264.7 cells; (**b**) ability of macrophages to phagocytize FITC-dextran. Values were expressed as mean ± SD obtained from triplicate experiments (*n* = 3). Statistical analysis was performed by an independent *t*-test using the statistical analysis software SPSS 11.5. ** *p* < 0.01 versus the negative control.

The effect of HPCG2 on the viability of RAW264.7 macrophages was investigated by MTT assay. As displayed in Figure 8a, HPCG2 had no obvious cytotoxic effect on RAW264.7 cells within the tested concentration range in vitro.

### 2.6. Effect of HPCG2 on the Phagocytic Activity of Macrophages

Phagocytosis of macrophages is a pivotal step in the immune response, which mediated the innate immune response to eliminate foreign substances. Thus, strengthening of macrophage phagocytosis is a key feature of macrophage activation [23]. In this study, the effect of HPCG2 on the phagocytosis of macrophages for FITC-Dextran was detected by flow cytometry, and the phagocytosis was expressed as the mean fluorescence intensity (MFI) of FITC-dextran engulfed by macrophages. When RAW264.7 cells were incubated with HPCG2 for 24 h, the MFI increased within the tested concentration range (Figure 8b). These results illustrated that HPCG2 significantly strengthened the phagocytic function of RAW264.7 cells.

### 2.7. Effects of HPCG2 on the Expression of CD86 and MHC II on RAW264.7 Cells 

**Figure 9:**
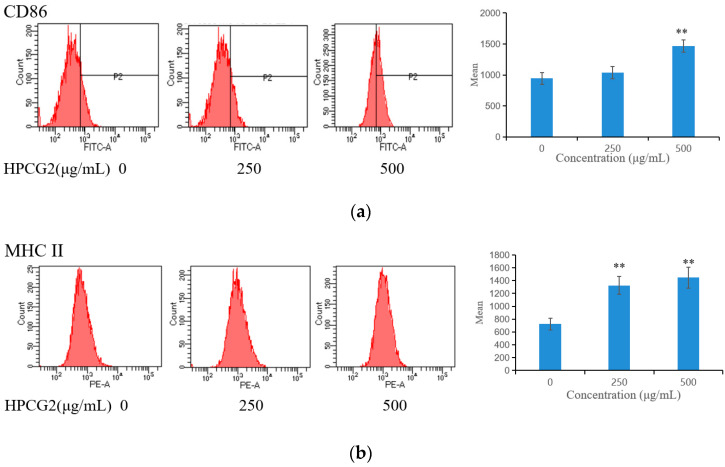
Effects of HPCG2 on expression levels of surface molecules in RAW264.7 cells. (**a**) CD86; (**b**) MHC II. The values are presented as mean ± SD (*n* = 3). ** *p* < 0.01 versus negative control.

The expression of CD86 and MHC II on RAW264.7 cells was measured by flow cytometry after treatment with different concentrations of HPCG2 for 24 h. HPCG2 increased the expression of M1 characteristic surface molecules CD86 and MHC II in a dose-dependent manner (Figure 9), which indicated that HPCG2 increased the proportion of M1 macrophages in RAW264.7 cells. M1 macrophages mediate host defense to infections and remove external invaders, exerting antitumor effects [24]. Thus, this result suggested that HPCG2 might enhance the defensive function of macrophages by inducing M1 polarization of macrophages.

### 2.8. Effect of HPCG2 on the Production of Nitric Oxide, IL-6, and TNF-α

**Figure 10:**
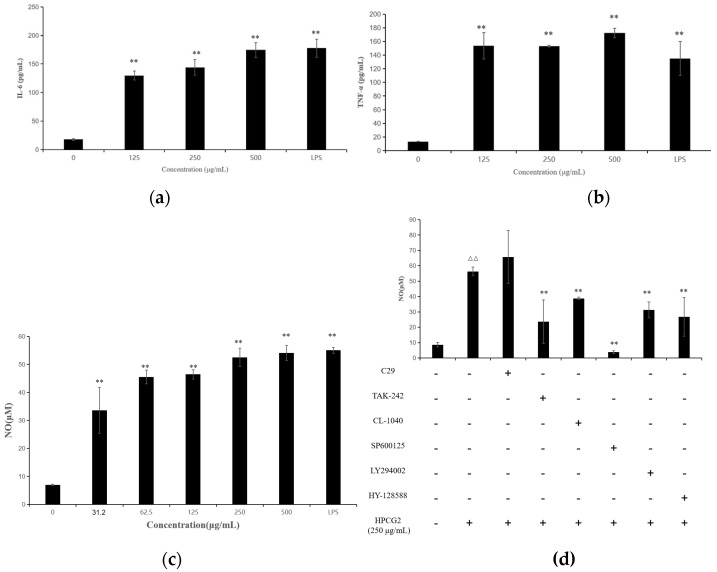
Effects of HPCG2 on the production of IL-6, TNF-α and nitric oxide in RAW264.7 cells. (**a**) IL-6; (**b**) TNF-α; (**c**) NO. Values are expressed as mean ± SD obtained from triplicate experiments (*n* = 3). Statistical analysis was performed by an independent *t*-test using the statistical analysis software SPSS 11.5. ** *p* < 0.01 versus the negative control. (**d**) NO secretion level of macrophages treated with different inhibitors. Values are expressed as mean ± SD obtained from triplicate experiments (*n* = 3). Statistical analysis was performed by an independent *t*-test using the statistical analysis software SPSS 11.5. ^ΔΔ^ *p* < 0.01 versus the negative control, ** *p* < 0.01 versus the model control.

M1 macrophages have been known to release pro-inflammatory cytokines, such as IL-6, TNF-α, and nitric oxide (NO), which exert a pro-inflammatory activity. IL-6 and TNF-α are the characteristic cytokines of M1 macrophages [25]. Figure 10a,b showed that HPCG2 led to markedly increased secretion of typical proinflammatory cytokines (TNF-α and IL-6) in RAW264.7 cells in a dose-dependent manner. In addition, NO is one of the signaling molecules related to macrophage cytolytic function [25]. As shown in Figure 10c, HPCG2 increased NO production in a dose-dependent manner, and the NO production of the RAW264.7 cells treated with HPCG2 (500 µg/mL) was similar to that of the positive control (1.00 µg/mL LPS). Therefore, HPCG2 could remarkably promote the secretion of M1 chemokines and toxic molecules, which was consistent with the expression of CD86 and MHC II on RAW264.7 macrophages after HPCG2 treatment. 

### 2.9. Effects of HPCG2 on the Expression of TLRs, Akt, MAPKs, and STAT3 in RAW264.7 Cells

**Figure 11:**
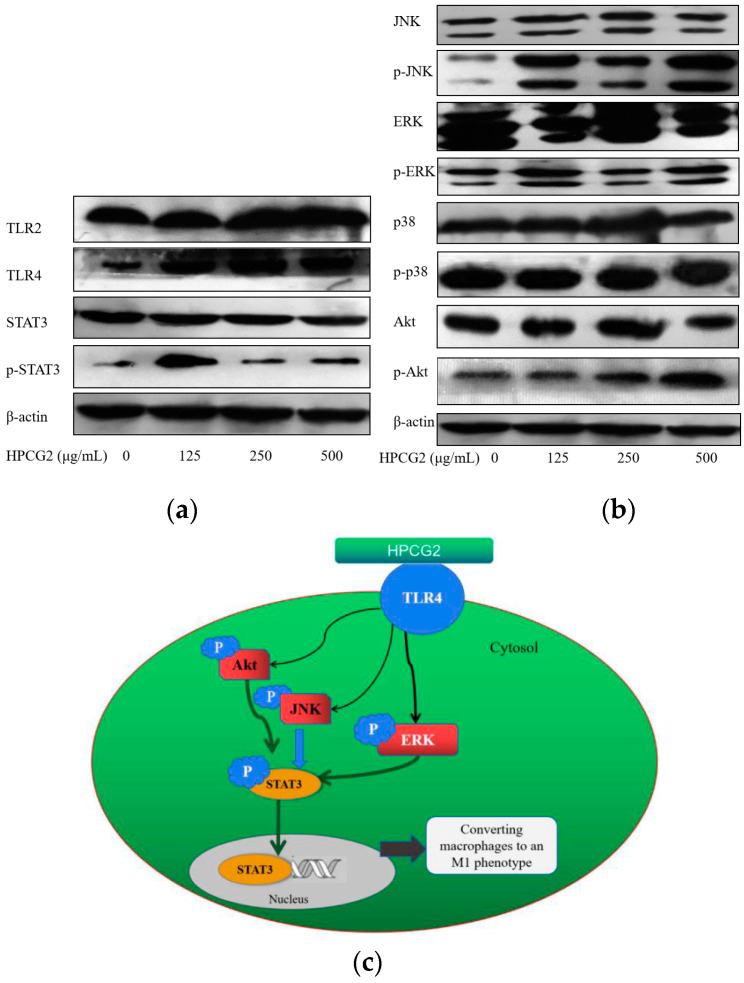
Effects of HPCG2 on expression of TLRs, Akt, MAPKs and STAT3. (**a**) effect of HPCG2 on the expression of the TLRs/STAT3; (**b**) effect of HPCG2 on the expression of MAPKs/Akt; (**c**) schematic illustrating the potential signaling pathways involved in macrophage polarization by HPCG2. The figure was representative of three independent experiments (*n* = 3).

The defensive mechanisms of macrophages can be activated, when various stimulus molecules bind to pattern recognition receptors (PRRs), such as toll-like receptors (TLRs) and C-type lectin receptors on the surface of macrophages, and touch off several different signaling pathways [26]. In general, proteins, polysaccharides and other macromolecules could not enter cells directly because of their large molecular mass. It has been reported that the first step for biomacromolecules to perform their roles is the recognition by PRRs. TLRs are one kind of important PRRs in immune cells, especially macrophages [27]. We examined the expression of TLR2 and TLR4 proteins by Western blot experiments. As shown in Figure 11a, HPCG2 upregulated the expression of TLR4, but not TLR2. Meanwhile, Figure 10d illustrated the following interactions: there was inhibition of NO secretion when the macrophages were pre-treated with TAK-242 (TLR4 inhibitor), but not C29 (TLR2 inhibitor). The results showed that the M1 polarization of macrophages induced by HPCG2 were probably associated with TLR4. 

It has been reported that Akt and three subunits of the mitogen-activated protein kinases (MAPKs), c-Jun N-terminal kinase (JNK), extracellular-signal-regulated kinase (ERK), and p38 participate or partially participate in the M1 polarization of macrophages. Meanwhile, Akt and MAPKs signaling pathways are downstream of the TLRs’ pattern recognition receptors [24]. Thus, we examined the effect of HPCG2 on the phosphorylation of Akt and three subunits of MAPKs in macrophages by Western blotting analysis. As shown in Figure 11b, HPCG2 promoted the phosphorylation of Akt, ERK, and JNK over the tested concentration range, but had no effects on the phosphorylation of p38, which indicated that Akt, ERK, and JNK participated in the polarization effects induced by HPCG2 on macrophages. Furthermore, the pre-treatment with LY294002 (Akt inhibitor), SP600125 (JNK inhibitor), and CL-1040 (ERK inhibitor) significantly suppressed the increase in NO production by HPCG2 (250 µg/mL). However, pre-treatment with SB203580 (p38 inhibitor) has no effect (Figure 10d). These results indicated that Akt, JNK, and ERK proteins were involved in the secretion of NO by macrophages induced by HPCG2. Akt, JNK MAPK, and ERK MAPK might also be in the M1 polarization of macrophage and phagocytosis [25,28]. 

It has been reported that STAT3 is a common transcription factor for the TLR4 signaling pathway. Phosphorylated STAT3 is translocated to the nucleus where it binds to DNA and promotes target genes transcription [29,30]. We found that the expression of p-STAT3 was upregulated after HPCG2 treatment (Figure 11a). Notably, Figure 10d also illustrated that the changes in secretion of NO induced by HPCG2 were blocked by HY-128588 (STAT3 inhibitor). All of these results showed that STAT3 was a transcription factor involved in the process of polarizing to M1 macrophages induced by HPCG2. Thus, we may deduce that the polarization of macrophages to be an M1 type by HPCG2 treatment may be related or partly related to the activation of TLR4, Akt, MAPKs, and STAT3 pathways. A schematic illustrating the potential signaling pathways involved in macrophage polarization by HPCG2 is presented in Figure 11c.

## 3. Discussion

Marine-derived compounds with an immunomodulatory effect have been extensively reported in recent years. Proteins and peptides were important research objects for the exploration of new immune modulators, and they were thought to work with different mechanisms and elicit multiple immune responses to eliminate pathogens [7]. A lectin (MTL) isolated from *Mytilus trossulus* exhibited an immune-enhancing effect by causing the increased expression of TNF-α and IFN-γ, and inhibited hyper-expressions of IL-10 [31]. Tachylectin from hemocytes of *Carcinoscorpius rotundicauda* was regarded as a pathogenic recognizing receptor [32]. In addition to lectins, keyhole limpet hemocyanin (KLH) isolated from *Megathura crenulata* has been shown to be effective in treatment with non–muscle-invasive bladder cancer because of its immune-stimulating effect on multiple lymphocytes [33]. *Fissurella latimarginata* hemocyanin (FLH) has shown to delay growth of B16F10 melanoma cells in mice by triggering a stronger humoral response than traditional hemocyanins [34]. The most widely discussed immune-related marine-derived protein compounds are antimicrobial peptides (AMPs). It was reported that some of them, such as clavanin-MO [35], Pt5 [36], and the defense from *Crassostrea virginica* [37] could regulate host immune levels in response to infection while inhibiting or killing microorganisms directly. These known compounds often act on the host’s immune system by promoting lymphocyte proliferation, increasing NK cell activity, or regulating cytokine production (anti-inflammatory or pro-inflammatory) [7]. As described in this study, HPCG2 was a new protein isolated from the hemolymph of *S. broughtonii* and was proven to be immune-enhancing *in vitro* on the immune cells. The immunomodulatory activity of HPCG2 suggested that it may play a similar role in humoral immunity of *S. broughtonii* and was likely to be a newly discovered immunoactive protein in the shellfish. 

Meanwhile, HPCG2 was classified into the CRISP-related family through sequence similarity searches and domain prediction. It was reported that protein members in this family were characterized by highly enriched cysteine and were widely distributed in nature [22]. Studies have shown that proteins of this family have certain immunomodulatory activities. Venom Ag5 proteins from wasp were shown to be potent allergens which cause a strong immune response in human body [38,39]. Pr-1 proteins distributed in plants were highly upregulated after pathogen infection. This process also occurred in uninfected parts of plants, which was thought to be a phenomenon of plant acquired immune responses [40,41]. In addition, the GLIPR1 subfamily in the CRISP-related family was also annotated to participate in immune or defense processes. Homologous with proteins from CRISP-related family, HPCG2 is likely to have immunomodulatory activities.

The spleen contains a large number of T- and B-lymphocytes and NK cells [2]. HPCG2 significantly promoted spleen lymphocyte proliferation, which demonstrated that HPCG2 could significantly activate T- and B-cells in the spleen and enhance the spleen involved immunity. HPCG2 could promote the proliferation of splenic lymphocytes, which illustrated that HPCG2 might have immunoregulatory activity for lymphocytes. Meanwhile, HPCG2 could promote NK cells to kill YAC-1 cells. NK cells play a major role in the resistance of viruses and tumor cells through their cytotoxicity and cytokine secretion. Except for the direct effect on antigens, NK cells could accelerate the activation, differentiation, and enlistment of other immune cells [42]. Therefore, HPCG2 might enhance lymphocyte and NK cell-mediated antitumor, antiviral infection, and immunomodulatory reaction.

Macrophages are one of the most important immune cells in the body, which play a vital role in innate host defense against external invaders and clear damaged cells. Meanwhile, macrophages may polarize into two different phenotypes, M1 or M2 type. M2 macrophages alternatively induced by Th2 cytokines (IL-4 and IL-13) act as a powerhouse for tumor angiogenesis and metastasis, by displaying an immunosuppressive phenotype and secreting abundant pro-tumor cytokines. M1 macrophages are classically activated by interferon [IFN]-γ. The secretion of TNF-α, IL-6, and NO are elevated by M1 macrophages, which are typically described as tumor-killing macrophages. M2 macrophages in the tumor microenvironment facilitate the immunological tolerance and tumor progression microenvironment, and most macrophages in the tumor microenvironment are M2 type [43]. TAMs-targeted therapy is a promising approach that could be used to reverse the immunosuppressive tumor microenvironment. Our results showed that HPCG2 could polarize macrophages to be M1 type at the level of cytokines and surface molecules, which suggested that HPCG2 might be involved in macrophage phenotype related diseases, such as tumors. This has not been reported in the study of marine proteins. Furthermore, our results illustrated that M1 polarization induced by HPCG2 was related to its activation of the TLR4-MAPKs-Akt-STAT3 signaling pathway. It has been reported that Akt and three subunits of MAPKs participate or partially participate in the M1 polarization of macrophages. MAPKs and Akt are the key regulators of pro-inflammatory factors [26]. Therefore, our research results showed that HPCG2 could polarize macrophages to be the M1 type from pharmacodynamics and signal pathways, which had not been reported in the current research of protein drugs. Therefore, for some diseases involving macrophage immune deficiency, such as tumors, HPCG2 might be developed as a potential immune treatment reagent.

## 4. Materials and Methods

### 4.1. Biological Materials

The experimental materials, *S. broughtonii*, were purchased from Chengyang seafood market in Qingdao, China, and were identified by Yu Rongmin (Jinan University, Guangzhou, China). The hemolymph fraction was collected and stored at −20 °C before processing. 

### 4.2. Reagents and Cultures

DEAE Sepharose fast flow, Phenyl Sepharose CL-4B and Sephadex G-75 were purchased from GE Healthcare (Shanghai, China). Tris and sodium dihydrogen phosphate, dimethylsulfoxide (DMSO) and 3-(4,5-dimethylthiazol-2-yl)-2,5-diphenyltetrazolium bromide (MTT) were obtained from Sigma Chemical Co. (St Louis, MO, United States). NO Griess reagent kit was obtained from Beyotime Corp (Shanghai, China). FITC anti-mouse CD86, FITC-dextran, and PE anti-mouse MHC II were obtained from Biolegend Corp (Shenzhen, Guangdong, China). DMEM medium, RPMI-1640 medium and fetal bovine serum (FBS) were purchased from Gibco Invitrogen Corp (San Diego, CA, USA). Murine TNF-α and IL-6 ELISA test kits were obtained from Excell Corp (Shanghai, China). ECL Western blotting detection kit was obtained from Tanon Crop (Shanghai, China). Anti-TLR2, anti-TLR4, anti-p-STAT3, anti-STAT3, anti-MAPKs, anti-p-MAPKs, anti-Akt, and anti-p-Akt primary antibodies were provided by Cell Signaling Technology (Beverly, MA, USA). All other reagents used in the experiments were of analytical grade.

RAW264.7 cell line and YAC-1 cell line were obtained from Cell Bank of Type Culture Collection of the Chinese Academy of Sciences (Shanghai, China) and were maintained in DMEM supplemented with 100 IU mL^−1^ penicillin, 100 μg/mL streptomycin and 10% FBS at 37 °C under humidified air with 5% CO_2_.

### 4.3. Purification Process 

Crude protein (HP) was extracted from the hemolymph and was sonicated using an ultrasonic cleaner (KQ-500E, 500W, Kunshan Ultrasonic Instruments Co., Ltd., Kunshan, China) for 40 min. The protein extract was surrounded by ice and the ice was replenished to ensure low temperature conditions. The supernatant was retained after the centrifugation (10,000 rpm, 30 min) and was precipitated with 100% ammonium sulfate (w/v) with stirring for 1 h. The crude protein was collected by centrifugation (10,000 rpm, 30 min) and dissolved in 0.03 M Tris-HCl buffer (pH 8.0), which was then dialyzed in the same buffer at 4 °C and lyophilized.

An anion exchange chromatography (DEAE Sepharose fast flow) was used as the first step in the purification process. Lyophilized crude protein was dissolved in 30 mL 0.1 M NaCl prepared in 0.03 M Tris-HCl buffer (pH 8.0), which was also used as an equilibration buffer. After equilibrium was reached, the crude protein was subjected and eluted stepwise with 0.1 and 0.5 M NaCl prepared in 0.03 M Tris-HCl buffer (pH 8.0). All the elution profiles were dialyzed in distilled water at 4 °C and lyophilized.

Then, the fraction HP2 was dissolved in 5 mL 1.0 M ammonium sulfate prepared in 0.03 M phosphate buffer (pH 8.0) and further applied to a Phenyl Sepharose CL-4B column and eluted stepwise with 1.0, 0.6, 0.3, and 0 M ammonium sulfate prepared in 0.03 M phosphate buffer (pH 8.0) and distilled water. All of the fractions were dialyzed in distilled water and lyophilized. The purified protein HPCG2 was finally isolated from fraction HP2C3 by Sephadex-G75 gel chromatography under the elution of distilled water and lyophilized. The absorbance at 280 nm was monitored through the whole purification process.

### 4.4. Purity Determination

SDS-PAGE analysis was used to separate proteins of different molecular weights and as a means of purity identification during separation [44]. HPCG2 was analyzed by the separation gel of 12% acrylamide concentration. Protein bands were shown by a Coomassie blue staining method [45].

The sample was loaded onto an ACE EXCEL HPLC column (250 × 4.6 mm, 5 μm) connected to Agilent series 1200 HPLC system. The solvent system was composed of distilled water and acetonitrile. The flow rate was 1 mL/min with a linear gradient of 0–100% acetonitrile within 30 min and 40–100% acetonitrile for 10 min, and then 100% acetonitrile was kept for 10 min. The detection wavelength was 280 nm, and the column temperature was 30 °C.

### 4.5. Measurement of Protein and Carbohydrate Concentration 

The protein concentration of HPCG2 was measured according to the procedure in the Bradford kit and bovine serum albumin (BSA) was used as the standard [46]. Determination of total sugar content in HPCG2 was performed using the sulfuric acid-phenol method with minor modification [47]. Glucose standard solutions of different concentrations were used to make a standard curve with the absorbance at 490 nm. The concentration of purified protein was 1.0 mg/ml. Each measurement was repeated three times.

### 4.6. Determination of Molecular Weight

The determination of molecular weight was carried out by ProteinGene Biotech Co., Ltd. (Wuhan, China). The sample was mixed with the CHCA matrix solution and then detected by a MALDI TOF/TOF high resolution mass spectrometer (MALDI TOF/TOF 5800, AB SCIEX) under positive ion mode. The raw data and map generated by the test were exported by DATA Explorer V4.5 software (Applied Biosystems, Waltham, MA, USA).

### 4.7. Elucidation of Secondary Structure

Infrared spectrum measurement of HPCG2 was carried out on an AP-2000 polarimeter (Jasco, Tokyo, Japan). The sample was fully dried in a dryer with P_2_O_5_, mixed with potassium bromide powder and then pressed into tablets.

CD analysis was used to determine secondary structure of HPCG2. The purified protein was dissolved in distilled water at 0.01 mg/mL and was measured at 20 °C by Chirascan plus circular dichroisms pectrometer (Applied Photo Physics Ltd., Leatherhead, Surrey, UK). The constituent parts of the secondary structure were calculated using the CDNN program. The analysis was carried out in triplicate.

### 4.8. Amino Acid Sequence of HPCG2

The purified sample was loaded into SDS-PAGE with a gel of 12% acrylamide concentration. The stained protein band was cut into tiny pieces and further washed with distilled water. Then, the gel was washed twice with 300 μL ammonium bicarbonate (25 mm), 50% acetonitrile, and 100% acetonitrile, and was dehydrated until it turned white. After adding 15–20 μL of trypsin (0.01 μg/μL), the gel was covered with 30–40 μL NH_4_HCO_3_ solution (50 mM, containing 10% ACN) at 37 °C overnight.

The digested peptide was dissolved in Nano-LC mobile phase A (0.1% formic acid, 2% acetonitrile/water) and desalted using ChromXP C_18_ pre-column (3 μm, 120 Å) on Ekspert nanoLC 415 system (SCIEX, Concord, ON, Canada). Then, the sample was separated by C_18_ reversed phase chromatographic column (75 μm × 15 cm, 3 μm, 120 Å, ChromXP Eksigent) with a gradient of mobile phase B (0.1% formic acid, 95% acetonitrile/water) increased from 8% to 38% within 30 min. Eluted peptides were immediately loaded into a Triple TOF 6600 system (SCIEX, Concord, ON, Canada) combined with Nano Spray III ion source (AB SCIEX, Framingham, MA, USA). MS survey scans were performed under the Information Dependent Analysis (IDA) mode and the data were collected and processed through PEAKS Studio 8.5 (version 8.5, Bioinformatics Solutions Inc., Waterloo, ON, Canada) [48]. MS/MS data of HPCG2 was retrieved and aligned in transcriptome sequence of *S. broughtonii* (accession number SRR3947651).

The similarity searches were performed with the PSI-BLAST program at the website of the National Center for Biotechnology Information (http://www.ncbi.nlm.nih.gov/blast). Signal peptide observation was performed by the SignalP 4.1 server (http://www.cbs.dtu.dk/services/SignalP-4.1/). Protein domain features of HPCG2 were predicted by InterProScan tool (http://www.ebi.ac.uk/interpro/) and the homology modeling of HPCG2 was completed by the I-TASSER server (https://zhanglab.ccmb.med.umich.edu/I-TASSER/), and the predicted structure was visualized by VMD.1.9.3. 

### 4.9. Cell Viability Assay 

RAW264.7 cells in logarithmic growth phase were trypsinized and adjusted to a concentration of 2 × 10^4^ cells per mL in DMEM medium. The cell suspension was added to a 96-well microplate and incubated for 12 h in advance. HPCG2 was formulated into a certain concentration gradient (31.2 to 500 µg/mL) by double dilution and added to the incubated cells in the well. The medium and 1 μg/mL lipopolysaccharide (LPS) were used as the blank and positive control, respectively. After incubation at 37 °C in a humidified 5% CO_2_ environment for 24 h, 20 µl combined MTT (5 mg/mL) was added and the absorbance at 570 nm was measured directly from the 96-well microplate.

The splenic lymphocyte proliferation was detected by the MTT method. After obtaining the spleen lymphocytes in vitro, the splenocytes were placed into 96-well plates at 5 × 10^6^ cells/mL in RPMI 1640 medium (FBS 10%) and treated with HPCG2 (31.2 to 500 µg/mL) or Con A. All splenocytes were incubated at 37 °C under 5% CO_2_ for 48 h, and then 20 µL of MTT (5 mg/mL) were added to each well. After 4 h, the supernatant was absorbed slowly and the formazan precipitate was solubilized in DMSO (200 µL per well). The splenic lymphocyte proliferation was measured by determining the optical density (OD) at 570 nm.

### 4.10. Secretion of Nitric Oxide and Cytokines

RAW264.7 cells (1 × 10^6^ cells per mL) were cultured in a 96-well plate for 12 h and then treated with series concentrations (31.2 to 500 µg/mL) of HPCG2. DMEM medium (FBS 10%) and LPS (1 μg/mL) were used as the blank and positive control, respectively. Secretion of NO was determined using the Griess method, where the nitrite accumulation was measured and NaNO_2_ was used as a standard for calculation. The synergistic effect of HPCG2 and different pathway inhibitors on NO production was also investigated. The pretreatment of macrophages with different inhibitors for 2 h was performed before adding HPCG2. The quantification of TNF-α and IL-6 secreted in the medium was performed using the ELISA kits.

### 4.11. Expression of CD86 and MHC II

When testing the phenotype of macrophages, RAW264.7 cells (1 × 10^6^ cells per well) were cultured in a 6-well plate for 12 h and then treated with series concentrations (250, 500 µg/mL) of HPCG2. The cell suspensions were hatched using the PE anti-mouse MHC II and FITC anti-mouse CD86 at 4 °C for 30 min. Ten thousand viable cells of every group were analyzed by a BD FACS flow cytometer (BD Biosciences, San Jose, CA, USA).

### 4.12. Phagocytosis Assay

Detection of FITC-dextran internalization was performed using flow cytometry for further verification of phagocytosis of RAW264.7 macrophages. Cells exposed to different concentrations of HPCG2 (250, 500 µg/mL) were collected and FITC-dextran (1 mg/mL) was added. After incubation at 37 °C for another 1 h, cold PBS was used to terminate the reaction. Cells were washed three times and re-suspended in PBS. The uptake of FITC-dextran in macrophages was detected by flow cytometry.

### 4.13. Western Blot Assay

RAW264.7 cells were incubated in the presence of HPCG2 (0, 125, 250, 500 μg/mL) for 24 h. The cells were collected and washed with cold PBS twice. Proteins were extracted with the RIPA and the concentration of each sample was determined by the BCA assay kit. Equal volumes of total protein and pre-stained protein ladder were separated by SDS-PAGE and then transferred into the 0.45 μm PVDF membrane. After blocking with 5% skimmed milk for 3 h, the membranes were then incubated with the primary antibodies overnight at 4 °C. After being washed three times with TBST, the membranes were incubated with the secondary antibodies and washed three times, and then the proteins were visualized using an ECL Western blotting reagent.

### 4.14. Natural Killer Cell Cytotoxicity Assay 

YAC-1 cells were used as target cells, and the splenic lymphocytes of mice were prepared as effector cells. The spleen lymphocytes (100 µL, 1 × 10^7^ cells/mL) were incubated for 48 h with different concentrations of HPCG2 (from 31.3 to 500 µg/mL). The same volume of target cells (2 × 10^5^ cells/mL) was then added to the wells of 96-well plates in the sample group. Notably, the wells that contained only effector cells or target cells were considered as effector and target cells control groups, respectively. Each step was carried out in triplicate. After 8 h of incubation, MTT (5 mg/mL) was added to each well. After 4 h, the supernatant was absorbed slowly and the formazan precipitate was solubilized in DMSO (200 µL per well). The OD value at 570 nm for each well was measured. The percentage of NK cell cytotoxicity was calculated from the following formula:

$$\mathrm{NK}\;\mathrm{cell}\;\mathrm{cytotoxicity}=\left[1\;-\;\frac{\mathrm{ODS}\;-\;\mathrm{ODE}}{\mathrm{ODT}}\right]\text{×}\;100\%$$

where ODS refers to the OD value of the test sample, ODE refers to the OD value of the effector cell control, and ODT refers to the OD value of the target cell control [49].

## 5. Conclusions

In summary, HPCG2, a new CRISP-related protein isolated from the hemolymph of *S. broughtonii*. HPCG2 is a monomeric, homogeneous protein comprised of 251 amino acids and has a molecular mass of 30.71 kDa. Moreover, HPCG2 was proved to possess the immunomodulatory effect on the murine immune cells. It exerted the immunomodulatory effect through the TLR4/JNK/ERK and STAT3 pathways in RAW264.7 cells. These findings revealed that HPCG2 might be developed as a potential immunomodulatory agent or new functional product from marine organisms.

## Figures and Tables

**Table 1:** Secondary structure composition of HPCG2.

Secondary Structure	α-Helix	β-Sheet	β-Turn	Random Coil
Ratio (%)	28.4	26.0	18.6	29.9

## Supplementary Materials

The following are available online at https://www.mdpi.com/1660-3397/18/6/299/s1, Table S1: The fragment sequences of HPCG2 identified by LC-MS/MS. Figure S1: SDS-PAGE profiles obtained in the purification process. Figure S2: Protein signature prediction of HPCG2 through the InterPro scan server. Figure S3: The FT-IR spectrum of HPCG2.
