# Supplementary material for: Purification and Characterization of a New CRISP-Related Protein from Scapharca broughtonii and Its Immunomodulatory Activity
Source: Mar Drugs. 2020 Jun 4;18(6):299. doi: 10.3390/md18060299 (PMC7344751; doi:10.3390/md18060299)
Supplement: Supplementary file 1 [file marinedrugs-18-00299-s001.pdf]

marine drugs

MDPI

Supplementary Materials

Table S1. The fragment sequences of HPCG2 identified by LC-MS/MS.

Peptide	10lgP	Mass	ppm	m/z	Area	Start	End
A.ATLSSHTTEEFNNAMLNR.C	101.65	2034.943	3.2	679.3237	9.98E+05	114	167
A.Q(-17.03)KEASDNKAEYMYQMK.T	108.31	1945.855	-2.6	649.6239	2.47E+05	183	230
A.YDAALATASQAWADKC(+57.02)DFVH.T	70.79	2239.001	2.2	747.3424	6.48E+02	231	290
A.VC(+57.02)GHYQQVVWAMSK.G	110.7	1691.791	20	846.9198	7.07E+05	417	458
A.C(+57.02)PNNC(+57.02)VSDSLC(+57.02)EHSF.G	64.96	1824.687	34.2	913.3818	1.24E+02	609	653
G.DTVENC(+57.02)QDMIDSVGEEYLC(+57.02)K.G	88.91	2403.987	12.9	802.3466	1.58E+03	672	731
A.GGGYGQAGFQC(+57.02)DK.A	93.64	1343.556	-0.1	672.7854	5.94E+05	732	770

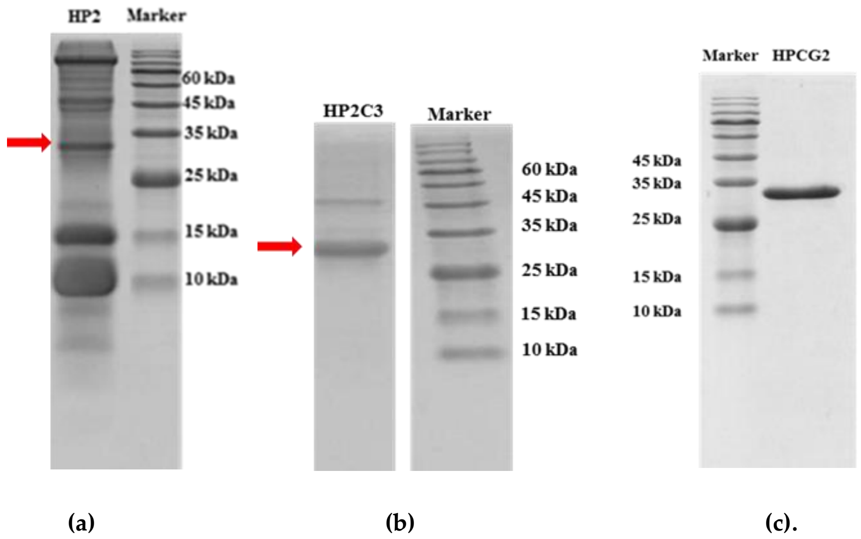

Figure S1. SDS analysis of profiles obtained in the purification process. (**a**) SDS analysis of profile HP2 eluted from DEAE Sepharose fast flow column; (**b**) SDS analysis of profile HP2C3 eluted from Phenyl Sepharose CL-4B column; (**c**) SDS analysis of profile HPCG2 eluted from Sephadex G-75 column.

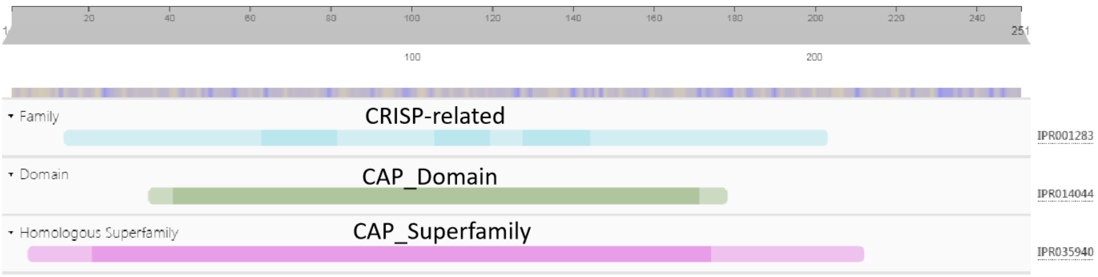

Figure 2. Protein signature prediction of HPCG2 through InterPro scan server.

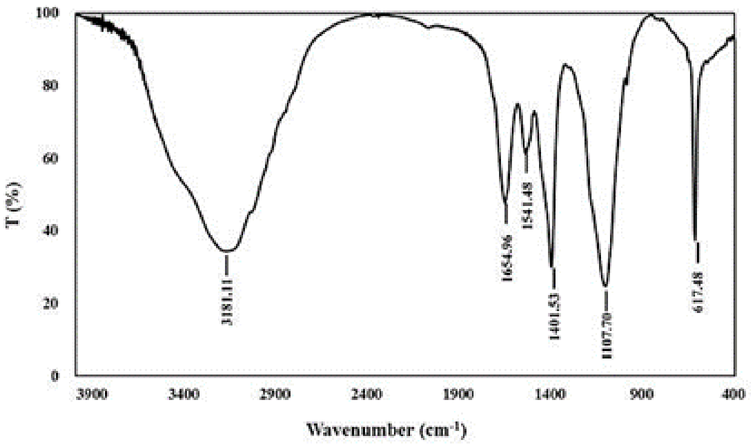

Figure 3. FT-IR spectrum of HPCG2.
